# Stress and Coping in Esports and the Influence of Mental Toughness

**DOI:** 10.3389/fpsyg.2020.00628

**Published:** 2020-04-23

**Authors:** Dylan Poulus, Tristan J. Coulter, Michael G. Trotter, Remco Polman

**Affiliations:** School of Exercise and Nutrition Sciences, Queensland University of Technology, Brisbane, QLD, Australia

**Keywords:** electronic sport, threat, challenge, control, intensity appraisal

## Abstract

This study explored stress and coping in electronic sports (esports) athletes and the influence of mental toughness (MT), as defined by two prominent conceptualizations: the 4/6Cs and Mental Toughness Index (MTI) frameworks. Participants were 316 esports athletes, ranked in the top 40% of one of five major esports: Defense of the Ancients 2, League of Legends (LoL), Counter Strike: Global Offensive, Overwatch and Rainbow Six: Siege. Participants completed the MTI, Mental Toughness Questionnaire 6 (MTQ6), Stress Appraisal Measure, and Brief COPE inventory. Results showed that MT (via both MT frameworks) was associated with perceived control, and MTQ6 subscales were associated with stress intensity. Mental toughness (both frameworks) was associated with the selection of more problem-focused and emotion-focused coping strategies and less avoidance coping strategies. The results indicate there is some overlap between the MT and stress-coping process in high-performing traditional sports and competitive esports athletes. These results suggest that esports athletes could benefit from sports psychology interventions designed for traditional sports athletes. Finally, the MTQ6 and MTI had low shared variance (20%), suggesting that the two questionnaires appear to measure different aspects of MT.

## Introduction

Electronic sports (esports) is the term used to describe the casual or organized playing of video games in a way that provides professional or personal development to the player (Pedraza-Ramirez et al., 2020). While there is still definitional debate around what is an esport and what is a video game, this study will be adopting the definition proposed by Pedraza-Ramirez et al. (2020). Esports is beginning to see increased attention from researchers (Reitman et al., 2019). One topic of interest and debate has been the comparison esports has with traditional sports (Bányai et al., 2018). This debate mainly centers on whether esports can be classified as a “sport” and if its players can be treated as traditional “athletes.” Jenny et al. (2017) identified that esports fitted well within the sociological and philosophical definitions of sport (e.g., it involves play, competition, and skill). Total prize pools for esports competitions are predicted to reach more than US $413 million by 2020 (Goldman Sachs, 2018). In 2019, the Defense of the Ancients 2 (DOTA 2) major tournament, “The International 9,” had a total prize pool of US $34 million, with the winning side (Team OG) collecting US $15 million (*The International, 2020*). Growth in viewership and prize pools has led to the development of professional players/teams competing in regular professional esports leagues (Taylor, 2012). Furthermore, talent development pathways for esports players are becoming more common with a number of governments and American (Conditt, 2016) and Australian student sporting portfolios (Lace, 2019) now recognizing esports athletes. As such, it would be important to examine some of the psychological factors that might determine success in esports.

On a psychological level, it has been suggested that the competitive and cooperative nature of esports requires similar mental skills as traditional sports (Murphy, 2009; Campbell et al., 2018). Supporting this idea, Himmelstein et al. (2017) qualitatively examined the mental skills and obstacles encountered by five League of Legends (LoL) athletes. To achieve optimal performance, 11 mental skills were identified (e.g., staying in the moment, utilizing preperformance routines, adapting to competition). Himmelstein et al. (2017) also identified four ways esports athletes acquired their skills (i.e., setting goals, analyzing performance, practicing individual skill, and maintaining a growth mindset). Smith et al. (2019), through interviews, investigated the stressors experienced and the associated coping strategies used by seven professional esports competitors. The associated coping strategies identified in the data supported the existing stress-coping literature (Polman, 2012). Emotion-focused (EFC), problem-focused (PFC), avoidance (AC), approach, and appraisal coping strategies were all employed by esports athletes (Smith et al., 2019). The studies by Himmelstein et al. (2017) and Smith et al. (2019) are limited by small samples sizes. Toth et al. (2019) explored esports players of different skill levels on tests of cognitive functioning. Results showed that elite esport athletes (based on in-game rankings) displayed faster response times and higher accuracy for simple choice reaction time stimuli (control trials), but that there were no differences between groups in cognitive inhibition. Clearly there is a need for more research on the psychological determinants of success for esports players (Pedraza-Ramirez et al., 2020).

A factor that has been shown to influence performance in sport is the way athletes cope with stressors they encounter (Lazarus, 2000). Considering esports has some similarities to traditional sport, it is timely to examine the stress and coping process in esports athletes to explore whether performance and well-being could be enhanced through established or new psychological interventions or training programs (Polman et al., 2018). An athlete’s ability to cope with stress has been shown to be important to success in traditional sport (Lazarus, 2000; Polman, 2012). The main framework adopted by researchers has been the cognitive–motivational–relational theory of stress and coping (Lazarus, 2000). According to this framework, appraisal of stressors, coping, and consequences are viewed as a dynamical and recursive process between the individual and his/her environment. Specifically, the person appraises events through primary (something at stake) and secondary (available coping options in relation to the event) appraisals (Lazarus, 2000). Following appraising an event as stressful, an individual invokes a voluntary coping response to manage the stress. Coping responses have been identified as falling into three common, higher-order dimensions (Nicholls and Polman, 2007): PFC (strategies aimed at changing stressful situation), EFC (strategies to regulate emotions associated with a stressful situation), and AC (physical or cognitive efforts to disengage from the stressor).

Studies have indicated that stable personality factors can directly or indirectly influence the stress-coping process (Polman et al., 2010). A disposition considered to be influential in sporting success is mental toughness (MT) (Coulter et al., 2018). There is ongoing debate around the conceptualization and definition of mental toughness (Gucciardi et al., 2015). This debate largely stems from two main perspectives that have recently emerged in the sport psychology literature.

The first perspective originated in Clough et al.’s (2002) work, who proposed the 4/6Cs model of MT. This model builds on Kobasa’s (1979) conceptualization of hardiness, a stress buffering personality trait. Based on interviews with coaches, athletes, and sport psychologists, Clough and colleagues added *confidence* (interpersonal and in one’s ability) to Kobasa’s (1979) three-factor hardiness construct: challenge, commitment (to one’s goals), and control (emotions and life). From this 4/6C model, Clough et al. (2002) developed a 48-item Mental Toughness Questionnaire (MTQ-48). While the MTQ-48 has been used extensively in the literature, several research teams have reported equivocal results about its psychometric properties (Gucciardi, 2017). To improve the factorial validity of the MTQ-48, Kawabata et al. (under review) recently refined the MTQ-48 to create a statistically and conceptually rigorous six-item multidimensional model of the 4/6Cs – the Mental Toughness Questionnaire 6 (MTQ6).

The second notable approach to the conceptualization and psychometric assessment of MT derives from qualitative methods to understand people’s perceptions of MT and its core attributes. Gucciardi et al. (2015) recently synthesized this body of research to identify several core properties of MT. Based on this work, the Mental Toughness Index (MTI) was developed (Gucciardi et al., 2015), which assesses seven core constructs: generalized self-efficacy, buoyancy, success mindset, optimistic style, context knowledge, emotion regulation, and attention regulation. According to Gucciardi et al. (2015), the MTI is a unidimensional measure that treats MT as a trait construct with state-like properties (i.e., it can fluctuate across time and context).

While similarities and differences exist across the two conceptualizations of MT, the current study aims to incorporate both perspectives into its research design, as an opportunity to explore the relationships and comparative explanatory impact the two perspectives have with stress and coping. In addition, the team-based nature of many esports requires its athletes to engage in interpersonal interactions. The 4/6C model (via the MTQ6) includes an interpersonal aspect to being mentally tough (interpersonal confidence), hence the decision to include it in the current study.

The relationship between MT and the stress and coping process has been researched in traditional sport. A study by Kaiseler et al. (2009) found that higher levels of MT were associated with lower levels of perceived stress and higher levels of emotional control. Furthermore, Nicholls et al. (2008) found that athletes who were more mentally tough used more PFC strategies and less AC strategies. Nicholls et al. (2012) also found that an athlete’s perception of challenge and threat was associated with one’s perceived control of the stressor. Higher levels of control were associated with perceiving the stressor as a challenge, and lower levels of control were associated with perceiving the stressor as a threat.

To better understand the relationship between MT and the stress and coping process in competitive esports players, this study will target players in the top 40% of their chosen esport. Esports games that use an in-game ranking system calculate players’ level of competence. The top 40% of competitors (according to in-game ranking) represents a large ability range and is more likely to capture players who play regularly and at a higher competency level. Esports players from five major team based esports will be selected in this study: DOTA 2, LoL, Counter Strike: Global Offensive (CS: GO), Overwatch (OW), and Rainbow Six: Siege (R6) (Pedraza-Ramirez et al., 2020). The games were chosen because of their popularity, prize pool (tournaments), in-game ranking system, and accessibility of participants (Goldman Sachs, 2018; Pedraza-Ramirez et al., 2020). Each of the five esports explored in this study have a competitive or ranked game play mode where an in-game ranking system sorts players into rankings based on their in-game competence.

The aim of the present study is to examine stress and coping in competitive esports athletes and explore how this regulatory process may be influenced by MT. To that end, it is predicted that esports athletes with higher MT scores will report lower levels of stress intensity and higher levels of perceived control, see stressors more as a challenge than a threat, and use more PFC and less EFC and AC (e.g., Nicholls et al., 2008, 2012; Kaiseler et al., 2009). Esports athletes who scored higher MT (total and item) will have higher levels of achievement (determined by in-game rank). Finally, the potential similarities or differences across the two MT conceptualizations were explored; no explicit *a priori* predictions were made.

## Methods

### Participants

Participants were 316 esports athletes (283 Males, 33 Female) aged 18–41 (*M* = 22.61, *SD* = 4.35). Table 1 provides descriptive statistics for gender, age, in-game rank, percentile group, and the frequency of professional players for each esports game. Table 2 shows the distribution of participants’ in-game ranks across each of their chosen esports and how in-game ranking was standardized and grouped into achievement levels across esports for statistical analysis.

**TABLE 1:** Frequency of gender, average age, average in-game rank, and frequency of professional players by esport game.

**Esport**	**Gender**		**Average age**		**Level of competition**		**Average in-game rank**
	**Male**	**Female**	**Mean**	***SD***	**Professional**	**Nonprofessional**	
DOTA 2 (*n* = 18)	18	0	21.67	4.20	2	16	Ancient 1
LoL (*n* = 118)	105	13	23.78	4.61	8	110	Platinum 2
CS: GO (*n* = 61)	56	5	21.38	3.60	16	45	Distinguished Master Guardian
OW (*n* = 84)	71	13	22.75	4.47	6	78	Diamond
R6 (*n* = 35)	33	2	21.00	3.05	8	27	Platinum 2
Total (*n* = 316)	283	33	22.61	4.35	40	276	-

**TABLE 2:** In-game ranking standardized into achievement level groups across esports.

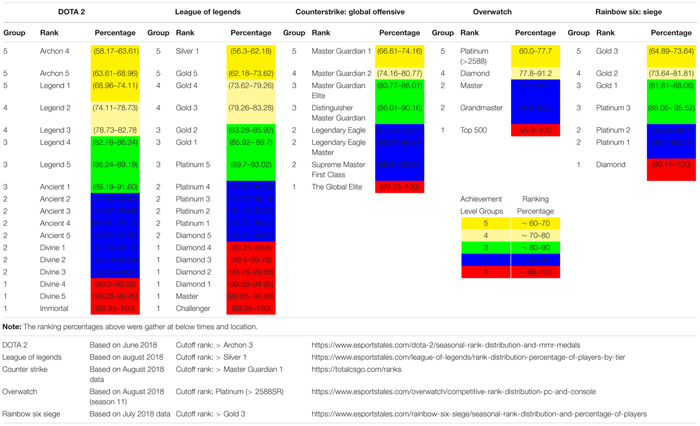		

### Measures

#### Demographics

The first part of the questionnaire pack for this study included completion of some demographic information including type of esport played, in-game ranking, age, and gender. The questionnaire allowed only players in the top 40% of their chosen esports (as determined by self-reported in-game rank) and players who played on a desktop computer, to continue through the questionnaire. In-game rank cutoffs were as follows: DOTA 2 ≥ Archon 3, LoL ≥ Silver 1, CS: GO ≥ Master Guardian 1, OW ≥ Platinum 1, R6 ≥ Gold3 (Table 2). No participants who met the in-game ranking cutoff were excluded. Players were also asked if they had ever competed in a professional esports tournament.

#### Stress Appraisal

To measure how esports athletes appraise stress, participants were asked to report a stressor that they recently encountered while playing their esport. On analog scales, participants rated the intensity of the stressor (Kowalski and Crocker, 2001) (1 = not stressful, 10 = extremely stressful), how much control they felt they had over the situation (1 = no control at all, 10 = full control), how much of a threat they felt the situation was (1 = not at all threatening, 10 = extremely threatening), and how challenging they felt the situation was (1 = not at all challenging, 10 = extremely challenging) (Kaiseler et al., 2012; Britton et al., 2019).

#### Coping

Coping was assessed by using the 28-item Brief COPE inventory (Carver, 1997). Using a four-point scale, the Brief COPE assesses how a participant has been dealing with the stressors in his/her life. The Brief COPE has 14 factors that can be classified into three higher-order dimensions: PFC, EFC, and AC coping (Dias et al., 2012). The Brief COPE has good psychometric properties (Carver, 1997). In the current study, reliability for 13 of the 14 factors was satisfactory (Table 3). Although one of the scales of the Brief COPE did not reach acceptable levels of internal consistency, it was included in the statistical analysis because estimates of internal consistency have limited applicability when assessing psychometric properties of measures of coping (Billings and Moos, 1981). Confirmatory factor analysis (CFA) was conducted, using AMOS 25, to explore the psychometric properties of the Brief COPE. This showed an excellent fit (CMIN/DF = 1.57, CFI = 0.95, RMSEA = 0.04, PCLOSE = 0.94).

**TABLE 3:** Mean, standard deviation, Cronbach α for stressor appraisal, coping strategies, mental toughness index, and mental toughness questionnaire.

**Stress appraisal**	**Mean**	***SD***	**α**
Perceived stress intensity	6.42	2.27	
Perceived control	3.86	2.92	
Perceived threat	4.07	2.97	
Perceived challenge	6.19	2.62	
**Coping strategies**			
**Problem-focused coping**	**2.47**	**0.63**	**0.81**
Active coping	2.79	0.79	0.72
Use of instrumental support	2.03	0.88	0.70
Positive reframing	2.42	0.92	0.81
Planning	2.65	0.83	0.70
**Emotion-focused coping**	**2.23**	**0.49**	**0.75**
Use of emotional support	1.99	0.89	0.83
Venting	2.06	0.81	0.63
Humor	2.67	0.83	0.84
Acceptance	2.99	0.78	0.60
Self-blame	2.39	0.90	0.72
Religion	1.29	0.63	0.71
**Avoidance coping**	**1.64**	**0.45**	**0.68**
Self-distraction	2.47	0.89	0.61
Denial	1.37	0.61	0.53
Substance use	1.19	0.47	0.87
Behavioral disengagement	1.53	0.71	0.68
**Mental toughness**			
**Mental Toughness Index (MTI)**	**5.23**	**0.95**	**0.86**
**Mental Toughness Questionnaire (MTQ6)**	**4.18**	**0.37**	**0.61**
**Challenge:** Challenges usually bring out the best in me.	4.16	0.67	
**Commitment:** I don’t usually give up under pressure.	4.29	0.63	
**Emotional control:** Even when under considerable pressure, I usually remain calm.	4.15	0.65	
**Life control:** I generally feel that I am in control of what happens in my life.	4.04	0.65	
**Confidence in abilities:** I am generally confident in my own abilities.	4.16	0.62	
**Interpersonal confidence:** I usually take charge of a situation when I feel it is appropriate.	4.33	0.62	

#### Mental Toughness

The two MT questionnaires administered were the MTI (Gucciardi et al., 2015) and the MTQ6 (Kawabata et al., under review). The MTI has eight items and is scored on a seven-point Likert scale. The MTI has shown good psychometric properties across a range of independent samples (Gucciardi et al., 2015) and measures overall MT. CFA for the present sample showed a good fit (CMIN/DF = 1.55, CFI = 0.99, RMSEA = 0.04, PCLOSE = 0.66).

The MTQ6 has six items and is scored on a five-point scale. Initial results have shown good factorial structure for the MTQ6 (Kawabata et al., under review). In the current study, CFA showed an adequate fit (CMIN/DF = 2.76, CFI = 0.92, RMSEA = 0.08, PCLOSE = 0.12).

### Procedure

The study received institutional ethical approval. Participants provided informed consent before participation in the study. Participants were recruited to the study through two methods, either online (*n* = 314) or in-person (*n* = 2) at esports events. Online participants were directed to a URL where they could complete the questionnaire pack (developed and managed by Qualtrics). The URL was distributed via email and online via social media (Twitter and Facebook) and YouTube.

### Data Analysis

Esports classify their players into levels, based on a percentage range. The number of levels differs across esports. To standardize in-game ranking across five esports into achievement level, five group classifications were developed: level 1 = 99–100%, level 2 = 90–98%, level 3 = 89–80%, level 4 = 79–70%, and level 5 = 69–60% (Table 2). Before the main analysis, a Shapiro–Wilk test was used to examine the distribution of each variable. All study variables were normally distributed, and skewness and kurtosis were not breached. Cronbach α’s and descriptive statistics were obtained for all study variables (Table 3). Pearson product–moment correlations between the variables were then calculated. Initial analysis compared differences between gender (Kaiseler and Polman, 2010) and game across stress appraisal, coping, and MT. No significant differences were found (all *P* > 0.05), and the data were collapsed across game and gender.

To determine whether MT was associated with perceived stressor intensity, stressor control, stressor threat, and stressor challenge, several separate linear regressions were run with the MTQ6 items (1–6), MTQ6 total, or MTI total, as predictor variables. To assess whether MT was associated with coping strategy selection, linear regressions were used. The 14 factors of the Brief COPE were entered as the dependent variables, whereas the MT measures represented the predictor variables. Similarly, regression analysis was conducted to explore the association between MT and coping at the dimensional level.

We first calculated Pearson product–moment correlations between the variable in this study. To investigate if achievement level was associated with participant’s stressor appraisal and selection of coping strategies, a number of multivariate analyses of variance (MANOVAs) were run. In the instance of significant main or interaction effects, follow-up analysis of variance (ANOVA) was conducted. Analyses of variance were conducted to explore if achievement level was associated with participants’ total MT levels. *Post hoc* comparisons were conducted using Sidak.

## Results

### Correlation Analysis of Study Variables

The means, standard deviations, and Cronbach α for stressor appraisal, coping strategies (dimensional and strategy level), and both MT questionnaires are reported in Table 3. The results of the correlational analysis of the study variables are shown in Table 4. Contrary to predictions, correlational analysis demonstrated that there was no association between overall MT level (both frameworks) and stressor intensity. However, there were small significant inverse relationships between MTQ6 dimensions, emotional control (*r* = -0.11), life control (*r* = −0.12), and stressor intensity. In terms of perceptions of control, only a small, significant positive correlation was identified with the MTI (*r* = 0.12).

**TABLE 4:** Correlational analysis of the study variables.

**Stress appraisal measure**	**Stressor**	**Stressor**	**Stressor**	**Stressor**	**Problem-focused**	**Emotion-focused**	**Avoidance**
	**intensity**	**control**	**threat**	**challenge**	**coping**	**coping**	**coping**
Stressor intensity							
Stressor control	−0.24*						
Stressor threat	0.44*	−0.19*					
Stressor challenge	0.48*	−0.13*	0.39*				
**Coping strategies**							
Problem-focused coping	0.08	0.21*	0.24*	35			
Emotion-focused coping	0.11*	0.04	0.19*	0.10	0.57*		
Avoidance coping	0.18*	–0.07	0.08	0.08	0.08	0.43*	
**MTQ6 total**	–0.08	0.05	0.07	−0.11*	0.26*	0.10	−0.20*
MTQ 1 – challenge	0.04	0.05	0.02	0.02	0.17*	0.07	0.02
MTQ 2 – commitment	–0.02	0.05	0.00	–0.09	0.18*	0.03	−0.22*
MTQ 3 – emotional control	−0.11*	0.05	−0.13*	–0.09	0.11	0.07	−0.15*
MTQ 4 – life control	−0.12*	–0.01	–0.07	−0.14*	0.09	0.04	–0.08
MTQ 5 – confidence in abilities	–0.01	0.00	–0.00	–0.05	0.17*	0.09	−0.12*
MTQ 6 – interpersonal control	–0.06	0.04	–0.07	–0.04	0.18*	0.06	−0.14*
**MTI total**	–0.06	0.12*	0.02	–0.01	0.35*	0.08	−0.27*
**Game rank**	0.16	0.03*	0.56	0.19	0.49	0.61	0.27

*p < 0.05.

For threat perceptions, a small significant inverse correlation with emotional control (*r* = −0.13) (MTQ6) was found, whereas for challenge perceptions, there was a negative correlation with MTQ6 total score (*r* = −0.11) and life control (*r* = −0.14). For PFC, there were small to moderate positive correlations with both the MTQ6 total (*r* = 0.26) and MTI (*r* = 0.35). In addition, small significant correlations were identified with challenge (*r* = 0.17), commitment (*r* = 0.18), confidence ability (*r* = 0.17), and interpersonal (*r* = 0.18) items of the MTQ6. Similarly, small significant inverse correlations were found between the MTQ6 total (*r* = −0.20), MTI (*r* = −0.27), and AC. The MTQ6’s commitment (*r* = −0.22), emotional control (*r* = −0.15), confidence ability (*r* = −0.12), and confidence (*r* = −0.14) interpersonal items also showed small significant negative associations with AC. Finally, there were no associations between MT (both frameworks) and EFC (all *P* > 0.05). See Table 4 for full results of the correlational analysis.

### Associations Between Coping Strategies and MT Measures

Regression analysis was run to explore the association between coping strategies and the six items of the MTQ, the MTQ6 total score, and MTI total score (Table 5). The PFC strategy, active coping, was associated with commitment (β = 0.16), confidence ability (β = 0.13), and interpersonal confidence (β = 0.13), explaining 11% the variance in the use of this strategy. Similarly, MTQ6 and MTI total scores respectively explained 9% (β = 0.30) and 15% (β = 0.38) of the variance in active coping. The use of instrumental support was only predicted by the MTI, which explained 2% of the variance in using instrumental support as a coping strategy. Higher levels of positive reframing and planning were also associated with higher levels of total MTQ6 (2%, β = 0.14; and 7%, β = 0.27) and MTI (4%, β = 0.20; and 10%, β = 0.31). Higher levels of challenge (β = 0.12) and interpersonal confidence (β = 0.17) also predicted increased use of the coping strategy planning (9%).

**TABLE 5:** Regression analysis to explore the association between mental toughness and coping strategies.

**Construct**	**Construct – MTQ6 (Items 1–6)**			**Construct – MTQ6 Total**			**Construct – MTI**		
			
**Coping strategies**	***R*^2^**	***F*(6, 309) =**	**Six MTQ 6 (1–6) – β significant predictors**	***R*^2^**	***F*(1, 314) =**	**Six MTQ 6 total – β significant predictor**	***R*^2^**	***F*(6, 309) =**	**Six MTI total – β significant predictor**
**Problem-focused coping**	0.07	4.02**		0.7	22.04**	0.26**	0.12	42.30**	0.36**
Active coping	0.11	6.45**	Q2 = 0.16**; Q5 = 0.13*; Q6 = 0.13*	0.9	30.96**	0.3**	0.15	54.16**	0.38**
Use of instrumental support	0.01	0.336		0.00	1.28		0.02	6.1*	0.14*
Positive reframing	0.02	1.16		0.02	6.09*	0.14*	0.04	13.18**	0.201**
Planning	0.09	5.19**	Q1 = 0.12*; Q6 = 0.17**	0.70	24.56**	0.27**	0.10	33.12**	0.31**
**Emotion-focused coping**	0.01	0.70		0.01	3.14		0.01	2.02	
Use of emotional support	0.02	1.21		0.01	2.79		0.01	2.54	
Venting	0.04	1.98		0.01	2.12		0.01	1.59	
Humor	0.02	1.24		0.01	3.24		0.00	0.41	
Acceptance	0.07	3.89**	Q1 = 0.13*; Q3 = 0.16**	0.05	18.88**	0.24**	0.02	5.81*	0.135*
Self-blame	0.01	0.64		0.00	0.02		0.00	0.03	
Religion	0.01	0.42		0.00	0.02		0.01	3.01	
**Avoidance coping**	0.08	4.63**	Q1 = 0.14**; Q2 = −0.20**	0.40	12.44**	-0.2	0.07	24.87**	-0.27**
Self-distraction	0.02	1.15		0.01	1.99		0.02	7.2**	-0.15**
Denial	0.02	1.19		0.01	1.71		0.00	1.37	
Substance use	0.04	2.33*	Q2 = −0.17**	0.20	5.9*	-0.16*	0.02	5.75*	-0.13*
Behavioral disengagement	0.10	5.43**	Q2 = −0.21**; Q6 = −0.16**	0.06	18.63**	-0.24**	0.12	42.84**	-0.35**

*p < 0.05; **p < 0.01.

Regarding EFC, acceptance was the only strategy associated with MT. In particular, the MTQ6 (5%, β = 0.24) and MTI (2%, β = 0.14) showed small but significant associations. In addition, challenge (β = 0.13) and emotional control (β = 0.16) (MTQ6) had a significant positive association with acceptance (7%). No associations were observed between the MTQ6 items, MTQ6 total, MTI, and the EFC strategies venting, humor, self-blame, or religion.

For AC, self-distraction (β = −0.15) was negatively associated with MTI total score (2%), whereas substance use and behavioral disengagement were negatively associated with both MTQ6 (2%, β = −0.16; and 6%, β = −0.24, respectively) and MTI (2%, β = −0.13; and 12%, β = −0.35) scores. Also, commitment (β = −0.17) (MTQ6) was associated with substance abuse (4%), whereas commitment (β = −0.21) and interpersonal confidence (β = −0.16) (MTQ6) were associated with behavioral disengagement (10%). There was no association between MT and denial. Table 5 provides details of the regression analysis.

### Influence of Achievement Level on MT, Stressor Appraisal, and Coping

Multivariate analyses of variance were used to test the influence of achievement level on MT, stressor appraisal, and coping (Table 6). Perceived stressor control was influenced by achievement level. Although there was a trend for higher-achieving players to perceive more control over the stressor, *post hoc* comparisons only showed that the 99th–100th percentile scored significantly higher than the 70th–80th percentile in levels of perceived control. There was no association between achievement level, perceived stressor intensity, threat, and challenge. Similarly, at both the strategy (Wilks’ λ = 0.80, *P* = 0.36, η*_p_*^2^ = 0.05) and dimensional (Wilks’ λ = 0.96, *P* = 0.45, η*_p_*^2^ = 0.01) levels, coping was not influenced by rank.

**TABLE 6:** MANOVA between achievement level and stressor appraisal, coping strategies, Mental Toughness Index, and mental toughness questionnaire.

**MANOVA**	**Wilks’**	***p***	**η*_p_*^2^**
Stress appraisal	0.91	0.74	0.02
Coping dimensions	0.96	0.45	0.01
Coping strategies	0.80	0.36	0.05
MTQ6 item (1–6)	0.86	0.17	0.04

**ANOVA**	***F*(4, 277)**	***p***	**η*_p_*^2^**

MTQ6 total	2.70	0.03	0.04
MTI	4.14	0.003	0.06

Achievement level was significantly influenced by MTQ6 items. *Post hoc* comparisons showed lower challenge scores for the 60th–70th percentile group compared to the 90th–99th and 99th–100th percentile groups. For commitment, the 80th–90th percentile group scored lower compared to the 90th–99th and the 99th–100th percentile groups.

One-way ANOVA showed significant differences between achievement level and both MTQ6 and MTI totals. *Post hoc* comparisons only showed that, for the MTI total, the 99th–100th and 80th–90th percentile participants scored higher than the 60th–70th percentile group.

There was a significant positive correlation between MTQ6 total and MTI (*r* = 0.45). The MTI positively correlated with all items of the MTQ6, except for life control. However, the shared variance between the MTQ6 and MTI was only 20% and lower between the items of the MTQ6 and MTI. See Table 7 for full results of the correlational analysis.

**TABLE 7:** Correlational analysis the mental toughness inventory, mental toughness questionnaire 6 total, and mental toughness questionnaire 6 subscales.

**Construct**	**MTQ6 total**	**MTQ 1 challenge**	**MTQ 2 commitment**	**MTQ 3 emotional control**	**MTQ 4 life control**	**MTQ 5 confidence in abilities**	**MTQ 6 interpersonal confidence**
**MTQ6 TOTAL**							
MTQ 1 – challenge	0.60**						
MTQ 2 – commitment	0.62**	0.33**					
MTQ 3 – emotional control	0.59**	0.24**	0.35**				
MTQ 4 – life control	0.52**	0.06	0.08	0.17**			
MTQ 5 – confidence in abilities	0.61**	0.19**	0.21**	0.17**	0.31**		
MTQ 6 – interpersonal control	0.57**	0.24**	0.20**	0.11	0.20**	0.28**	
MTI total	0.45**	0.33**	0.41**	0.18**	0.10	0.25**	0.30**

**p < 0.05.

## Discussion

The main purpose of this study was to examine stress and coping in esports athletes and explore how this regulatory process is influenced by MT. Results suggest that the MTI was associated with perceived control, and MTQ6 subscales were associated with stress intensity. Furthermore, MT was associated with how stress was perceived as being a challenge or threat (both inversely) and the selection of coping strategies.

### Association Between Stress Appraisal and MT

Results did not support the *a priori* prediction that esports athletes with higher overall levels of MT would report lower levels of stress intensity and higher levels of stress control. This observation contradicts previous findings by Kaiseler et al. (2009) and Levy et al. (2012), who found that higher levels of MT, using the MTQ-48, were associated with lower levels of perceived stress. This result might be explained due to esports athletes experiencing different types of stressors to traditional sports athletes (Nicholls and Polman, 2007). For example, esports athletes in the present study reported technical issues and antisocial behavior as stressors that have not been reported previously, however, further research is needed to understand this. Providing partial support for this study’s prediction, small significant negative associations were observed between emotional control and life control and stress intensity, indicating that esports athletes, with higher levels of emotional control, rated the intensity of the self-reported stressor lower. It appears that only those esports athletes, who reported to have higher levels of emotional control, were able to reduce the intensity of the perceived stressor.

In support of the initial hypothesis, a positive association was observed between overall MT (MTI) and stress control. Similar to previous findings (Kaiseler et al., 2009; Levy et al., 2012), those esports athletes higher in MT (MTI) reported more control over the self-reported stressor. Literature on MT has described more mentally tough people as having an unshakable faith in their abilities to control their own destiny and an increased ability to remain in control under pressure (Clough et al., 2002; Nicholls et al., 2008). The result here appears to match this description of mentally tough people and suggest that more mentally tough esports athletes have increased levels of perceived control over a stressor.

The present study did not support previous findings (Kaiseler et al., 2009; Levy et al., 2012) and the *a priori* prediction that MT would be associated with lower threat and higher challenge appraisal. There was only a small negative correlation between emotional control and threat perception. In addition, overall score of the MTQ6 and the life control subscale had a negative association with challenge. Previous studies have found athletes with higher overall MT perceive stressors more as a challenge than a threat (Nicholls et al., 2012). First, the present findings suggest that the relationship between challenge and threat appraisal is not dichotomous (Britton et al., 2019). Esports athletes appear to appraise stressors as both a threat and challenge at the same time. This finding might be explained through the use of a single item to measure threat and challenge. Second, differences in threat and challenge perception between esports and traditional sports athletes could be explained due to the online nature of esports. Nonprofessional play is largely done online through ranked or competitive play; players may not be able to choose all their teammates. Players are often randomly grouped with different teammates (this varies between esports), and the only outcome at stake is their in-game rank. When playing in a professional tournament, often in front of a live audience for a cash prize, the stakes could be comparable to traditional sports. This study, having less professionals (*n* = 40) than nonprofessional (*n* = 276) players, could account for the difference in threat and challenge perceptions.

### Associations Between Stress Coping and MT

Correlational and regression analysis showed support for the hypothesis that PFC, at both the dimensional and strategy levels (active coping, use of instrumental support, positive reframing, and planning), was positively associated with total MT (both frameworks). Higher levels of the MTQ6 subscales commitment, confidence in abilities, and interpersonal confidence predicted increased active coping, and higher levels of challenge and interpersonal confidence predicted increased planning. These results are consistent with previous research, which has shown that mentally tough athletes are more likely to use PFC strategies, suggesting that esports athletes in the top 40% cope with stressors similarly to high-performing sports athletes and that mentally tougher esports athletes appear to want to actively deal with their stressors (Nicholls et al., 2008).

Like previous research, mentally tougher esports athletes reported less use of AC and AC strategies (e.g., self-distraction, substance use, and behavioral disengagement; Nicholls et al., 2008; Kaiseler et al., 2009). Such associations were also observed for some of the factors of MT (MTQ6). Specifically, higher levels of commitment were associated with less substance use and behavioral disengagement, and interpersonal confidence with less use of behavioral disengagement. This finding suggests that athletes who have lower levels of MT, and who employ more AC strategies, may be less skillful and may not perform as well as athletes with higher levels of MT. These results also suggest that competitive esports and sports athletes with high levels of MT (both frameworks) cope similarly by employing less AC strategies.

Contrary to the hypothesis, MTI, MTQ6 total, challenge, and emotional control all positively predicted the use of acceptance (EFC strategy). This observation suggests that acceptance could be important for competitive esports athletes. The use of acceptance could be explained through the match-making algorithm used in solo queue. When playing ranked or competitive play, many factors can be out of the player’s control, which includes teammates, opponents, and character selection. Although these issues might result in stress, being able to accept that these factors are beyond a players control could be associated with performing more highly in esports.

Because this is the one of the first studies exploring stress and coping in an esports population, scores obtained for the Brief COPE were compared to those obtained in the sport domain. To this end, Dias et al. (2010) explored coping in a team sport setting using the Brief COPE. At the dimension level, traditional sports athletes reported the use of more PFC and EFC, and on a strategy level, they reported a higher use of instrumental and emotional support. Such differences between traditional sport and esports might be due to the fast-paced nature of esports. As such, esports athletes might have less time to invoke PFC strategies. Also, esports athletes used more acceptance and self-distraction strategies compared to the team athletes in Dias et al. (2010) study. These differences could be explained by the online nature of esports requiring acceptance or ignoring of stressors to perform at the highest level.

### Associations Between MT and Achievement Level

The current study found partial support for the notion that achievement level was associated with MT. In particular, those with higher ranks tended to have higher total and subscale MT scores, with a number of significant differences for challenge and commitment (MTQ6). Similar to traditional sport, these findings suggest there could be an association between esports performance and MT levels (Cowden, 2016). It would appear that those who are more successful esports athletes have higher levels of MT.

### Similarities and Differences Between the MTQ6 and MTI8

There were interesting similarities and differences that emerged from the results of the MTQ6 and MTI. Regression analysis shows that both MT measures predicted the use PFC and AC at a dimensional level, and active coping, planning, acceptance, substance use, and behavioral disengagement at a strategy level. Correlational analysis from the MTQ6 and MTI showed differences in stress appraisal. The MTQ6 was associated with perceived stressor intensity, threat, and challenge, whereas the MTI was associated only with perceived stressor control. Mixed results are not surprising considering each questionnaire represents a different, yet partly similar, framework. Furthermore, correlational analysis showed that the MTQ6 and MTI had low shared variance (20%). This would suggest that the two questionnaires measure different aspects of MT. Overall, these findings represent the ongoing debate surrounding the conceptualization of MT.

### Practical Implications

While further research is needed, the findings of the present study could be beneficial for sport psychologists working with esports athletes. Interventions to increase emotional control may help lower perceived stress intensity, potentially improving performance and quality of life. Furthermore, results suggest that acceptance coping is an important strategy used by esports players. Esport athletes who more effectively utilize acceptance coping may better deal with stressors caused by factors outside of their control (i.e., teammates and opponents). Based on the notion the esports athletes are more likely to report the use of PFC strategies and less AC strategies, it would be suggested that coaches, team managers, and/or sport psychologists help their athletes to actively deal with the stressors they experience, although future research should examine coping effectiveness.

### Limitations and Future Research Direction

The present study is not without limitations. A cross-sectional design was used, meaning that causality cannot be inferred. The constructs were measured using self-reported questionnaires. Data collected were retrospective and collected from players only in the top 40% of their esport, which limits the generalizability of the findings. Moreover, stress appraisal was assessed in relation to one specific stressful event, and the potential baseline differences in stress reactivity were not controlled for. One coping strategy measured by the Brief COPE showed low reliability, however, it was included in the analysis because previous research has indicated that estimates of internal consistency have limited applicability when assessing psychometric properties of measures of coping (Billings and Moos, 1981). Standardizing rankings across games means that the study could not control for differences in skill distribution or game difficulty between esports. Future research could investigate the relationship between MT and stress coping in one game and across all game ranks (Pedraza-Ramirez et al., 2020). In addition, it could be beneficial to explore how esports athletes cope over time and across multiple stressful events.

## Conclusion

The present study suggests that MT may influence the stress and coping process in esports athletes. There is an overlap between the MT and stress-coping processes in traditional sports and esports athletes. These similarities appeared more in the selection of coping rather than the appraisal process. This result suggests that esports athletes could benefit from traditional sports psychology interventions in MT and stress coping and that further research is required into the psychological determinants of success for esports athletes. Finally, low correlations between the MTQ6 and MTI – representing two of the fundamental and most popular models of MT in current literature – indicate that further debate is encouraged on how best to conceptualize MT.

## Data Availability Statement

The datasets generated for this study are available on request to the corresponding author.

## Ethics Statement

The studies involving human participants were reviewed and approved by the Queensland University of Technology Office of Research Ethics and Integrity. The patients/participants provided their written informed consent to participate in this study.

## Author Contributions

The study was designed by DP, TC, and RP. The data were collected by DP and MT. The data were analyzed by DP and RP. The manuscript was written by DP, TC, and RP. All authors read and approved the manuscript.

## Conflict of Interest

The authors declare that the research was conducted in the absence of any commercial or financial relationships that could be construed as a potential conflict of interest.
